# “I wouldn’t need Narcan for myself, but I can have it for somebody else:” perceptions of harm reduction among hospitalized patients with OUD

**DOI:** 10.1186/s13722-023-00395-w

**Published:** 2023-06-24

**Authors:** Rachel French, M. Holliday Davis, Shoshana V. Aronowitz, Molly Crowe, Matthew Abrams, Grace Edwards, Margaret Lowenstein

**Affiliations:** 1 National Clinician Scholars Program, University of Pennsylvania, 13th Floor Blockley Hall, 423 Guardian Drive, Philadelphia, PA 19104 USA; 2 Leonard Davis Institute for Health Economics, University of Pennsylvania, 3641 Locust Walk, Philadelphia, PA 19104 USA; 3 Perelman School of Medicine, University of Pennsylvania, 423 Guardian Drive, Philadelphia, PA 19104 USA; 4 School of Nursing, University of Pennsylvania, 418 Curie Boulevard, Philadelphia, PA 19104 USA; 5 College of Arts and Sciences, University of Pennsylvania, 249 S 36th St, Philadelphia, PA 19104 USA; 6 Research Director, Center for Addiction Medicine and Policy, University of Pennsylvania, Philadelphia, PA USA

**Keywords:** Opioid use disorder, Substance use disorder, Harm reduction, Hospitalization, Naloxone, Fentanyl test strips, Addiction, People who use drugs, Patient-centered care, Syringe service programs

## Abstract

**Background:**

Extant literature is limited on adoption of evidence-based harm reduction strategies in hospitals. We explored patient perceptions of incorporating harm reduction supplies and education in hospital care with patients with opioid use disorder (OUD).

**Methods:**

Qualitative descriptive study of hospitalized patients with OUD in Philadelphia, PA using semi-structured interviews conducted between April and August of 2022.

**Results:**

Three major themes emerged from 21 interviews with hospitalized patients with OUD: (1) Applicability and Acceptability of Harm Reduction Practices for Oneself; (2) Applicability and Acceptability of Harm Reduction Practices for Others; (3) Perceptions of Harm Reduction Conversations. Most participants were familiar with harm reduction but varied in their perceptions of its relevance for their lives. We noted differences in how participants viewed the applicability and acceptably of harm reduction practices that they perceived as intended to help others (e.g., naloxone) versus intended to help themselves (e.g., syringes). Most participants reported that meaningful conversations about drug use did not happen with their care team but that these conversations would have been acceptable if they were conducted in a way consistent with their individual substance use goals.

**Conclusions:**

Patients' interest and perceived acceptability of harm reduction services during hospitalization varied by individual patient factors and the perceived user of specific interventions. Given their positive potential, harm reduction practices should be incorporated in hospitals, but this must be done in a way that is acceptable to patients. Our findings reveal ways to integrate concepts from a harm reduction approach within a traditional medical model. More work is needed to understand the impact of such integration.

**Supplementary Information:**

The online version contains supplementary material available at 10.1186/s13722-023-00395-w.

## Background

Core to the harm reduction movement is the grassroots and community-based nature of the work. People who use drugs (PWUD) and people in recovery have long been at the forefront of harm reduction and continue to lead these efforts [1]. The philosophy of harm reduction seeks to elevate the complex and varied experiences of PWUD and has been defined as “a set of practical strategies and ideas aimed at reducing negative consequences associated with drug use” [2]. A harm reduction approach to caring for patients with substance use disorders (SUDs) emphasizes the rights and autonomy of PWUD, with the goal of mitigating substance-related harms and improving health without mandating abstinence [3]. Harm reduction approaches are useful for many PWUD, regardless of what substances they use and how they use them [4].

Substantial evidence points to the effectiveness of a broad range of harm reduction interventions. Examples of harm reduction strategies include naloxone, fentanyl test strips, syringe service programs, overdose prevention sites, and safe supply. Naloxone distributed by community-based organizations has been shown to be 75–100% effective at preventing mortality when administered to someone experiencing an overdose [5]. People who use syringe service programs contract HIV and hepatitis C 50% less [6], are five times more likely to enter substance use treatment [7], and are three times more likely to stop using substances than those who do not access such programs [7]. Evidence also suggests that harm reduction interventions like syringe service programs, medications for opioid use disorder, and HIV treatment are both cost-effective and cost-saving to society [8]. Overdose prevention sites enhance access to primary care and treatment for SUDs [9] while decreasing overdose mortality [10]. Despite strong evidence for the efficacy of harm reduction strategies, such approaches are severely underused [11].

Alongside the surging overdose crisis in the United States, rates of hospitalization for opioid use disorder (OUD) have increased over three-fold since 1998 [12], with emerging evidence also noting dramatic increases in stimulant-related hospitalizations [13, 14]. Weighted estimates from the National Inpatient Sample report 506,155 hospitalizations for OUD from 2016 to 2019 [15]. Despite the prevalence of PWUD in hospitals, many of these patients do not receive evidence-based SUD treatment during their hospitalization [16], resulting in poor outcomes. PWUD, compared to people who don’t use drugs, have higher rates of patient-directed discharge (also known as discharge against medical advice) [17]. They also have higher rates of readmission and post-discharge mortality, often related to untreated drug use [2, 17]. Among Oregon Medicaid patients with OUD who died within 12 months of hospital discharge, 58% of deaths were attributed to drug-related causes, including 13.6% attributed to overdose [18].

When evidence-based OUD care is provided to hospitalize patients, it is often focused on treating withdrawal and initiating medications like methadone or buprenorphine [4], an improvement in care thanks to notable efforts to decrease barriers to such medications in recent years [19]. While this care is highly effective for many patients with OUD, it fails to meet the needs of the many hospitalized patients who will continue to use substances following discharge [4, 20–22]. These approaches may also lack offerings for patients with SUDs that are not as effectively managed with pharmacotherapy (e.g., methamphetamine use disorder, cocaine use disorder) but would benefit from harm reduction supplies and education [4]. Some hospitals have capitalized on the opportunity to offer patients harm reduction tools during hospitalization [4, 23], but too often hospitalization continues to be a missed opportunity to meaningfully engage patients, especially those who are not interested in, ready for, or able to take medications for OUD [24, 25].

There is currently limited research focused on the adoption of evidence-based harm reduction strategies in hospitals and the exploration of the experiences and opinions of the end-users of such care: hospitalized patients with a history of or current substance use. In this study, we sought to understand attitudes of patients with OUD towards the incorporation of harm reduction supplies and education into hospital care and the acceptability of such practices. We also sought to explore a range of individual preferences around how to meet the harm reduction-related needs—including education and provision of harm reduction supplies—of hospitalized patients with OUD.

## Methods

We conducted a qualitative descriptive study using one-on-one, semi-structured interviews with hospitalized patients with OUD. The results we report in this paper focus on data collected from a subset of the questions on the interview guide that specifically addressed perceptions about harm reduction in hospital settings (see Table 1 for these interview guide questions). The larger study was focused on transitions of care for hospitalized patients with OUD. All participants were hospitalized at one of three Penn Medicine Hospitals in downtown Philadelphia, Pennsylvania: The Hospital of the University of Pennsylvania, Penn Presbyterian Medical Center, or Pennsylvania Hospital. At the time of the study, none of these hospitals had an addiction consult service, though this work helped to inform implementation of a consult service in one of these hospitals that launched in March 2023. At the time of the study, these hospitals had a inpatient peer support model, though this model was not explicitly rooted in harm reduction. We identified potential participants using an algorithm designed within Penn Medicine that is used as part of routine clinical care to identify patients likely to have OUD based on criteria extracted from their medical record [26]. Criteria include: receipt of buprenorphine, methadone, or naloxone while hospitalized; chief complaint consistent with OUD; or OUD diagnosis within the past year. Patients meeting these criteria populate a list in the electronic health record used for clinical and quality improvement efforts. This list was reviewed by research staff to guide study recruitment. A research assistant then approached potential participants during their hospitalization to screen for inclusion. Eligibility criteria included: (1) currently hospitalized, (2) currently having OUD based on Penn Medicine algorithm; (3) being at least 18 years old; and (4) being able to communicate fluently in English. We limited inclusion criteria to patients with OUD instead of all patients using substances because (1) this was a secondary analysis of another study focused on care transitions and medication for OUD initiation and continuity and (2) Penn Medicine currently has a screening algorithm to identify OUD but not other substance use disorders. Given that the algorithm to identify OUD does not guarantee current OUD, participants did not need to report active opioid use to be eligible.

**Table 1:** Interview guide about harm reduction

Are you familiar with the term harm reduction?
If people aren’t familiar, clarify that harm reduction could include anything that reduces negative consequences of drug use, like having naloxone on hand, never using alone, using clean needles, etc
Tell me about any experiences when your care team has discussed harm reduction during your hospital stay
Probe: Were these positive or negative experiences?
How would you feel about harm reduction tools being given to you during the hospital stay or when you are discharged?
What harm reduction tools would you find most useful?

We conducted interviews between April and August of 2022. After confirming eligibility and obtaining informed consent, interviews were conducted in-person in a private room in the hospital by trained research assistants (MHD, MC). MHD has a background working in community-based harm reduction and MC is a medical student with clinical and research experience working with patients with OUD. The study team members who conducted the interviews were trained by a senior researcher with experience in qualitative methods who is also an Addiction Medicine physician (ML). Interviews lasted approximately 30–60-min, and we collected demographic information following each interview. Participants were compensated with a $25 gift card upon interview completion.

Interviews were audio-recorded, de-identified, transcribed verbatim via a professional transcription service. Using NVivo (version 11.7) for qualitative data management, two members of the study team (MHD, RF) analyzed the findings using thematic content analysis [27]. Eighteen percent of the transcripts were double-coded with strong agreement among coders (κ = 0.8). We employed an inductive approach to keep codes close to the text. Accordingly, codes were grouped into four themes. Consensus of the themes was reached between the authors (MHD, RF, ML) after thorough discussion. We have followed the COREQ checklist for reporting on qualitative research (Additional file 1: COREQ checklist) [28].

The University of Pennsylvania Institutional Review Board provided ethical approval for this study (Protocol number: 849754). Information from interviews was kept on a secure server at the University of Pennsylvania. None of the information collected was shared with clinical staff and the data were de-identified for analysis.

## Results

We interviewed twenty-one hospitalized patients with OUD. The mean age of participants was 45 years (Table 2). Nearly one-third of participants were female (29%). Two-thirds of participants identified as white (67%). Nearly one in five participants were Latinx (19%), with about a quarter of participants identifying as Black (24%). Forty-three percent of participants were hospitalized for infections such as bacteremia, osteomyelitis, sepsis, and/or cellulitis. About a quarter (24%) had a primary diagnosis not directly related to drug use. Most participants (95%) currently used opioids, with about a third also reporting current use of stimulants and/or sedatives and 14% reporting current alcohol use. All participants were using at least one substance.

**Table 2:** Participant characteristics

Characteristic, n (%)	Study group (n = 21)
Age	
Mean (SD)	45 (11)
Gender	
Female	6 (29)
Male	15 (71)
Ethnicity	
Hispanic or latino	4 (19)
Race^a^	
White	14 (67)
Black	5 (24)
Unspecified other race	2 (10)
Hospital	
Penn Presbyterian Hospital	12 (57)
Hospital of the University of Pennsylvania	5 (24)
Pennsylvania Hospital	4 (19)
Primary diagnosis grouping	
Bacteremia, osteomyelitis, sepsis, and/or cellulitis	9 (43)
Non-drug use related condition	5 (24)
Wound-related condition	4 (19)
Withdrawal or overdose-related condition	3 (14)
Drugs used in last 6 months^a^	
Opioids	20 (95)
Stimulants	7 (33)
Sedatives	7 (33)
Alcohol	2 (24)
Other	3 (14)
Types of opioid used (n = 20)^a^	
Fentanyl	15 (75)
Heroin	13 (65)
Pressed pills	4 (20)
Prescription pills	4 (20)

^a^These characteristics total > 100% as participants could select all that apply

The study team identified three major themes by grouping codes: (1): applicability and Acceptability of Harm Reduction Practices for Oneself; (2) Applicability and Acceptability of Harm Reduction Practices for Others; (3) Perceptions of Harm Reduction Conversations. Below we detail each theme and provide illustrative quotations, with the parenthetical number following each quote representing the anonymized patient identifier we assigned to each participant.

### Theme 1: Applicability and Acceptability of Harm Reduction Practices for Oneself

When asked about their understanding of and prior knowledge about harm reduction, most participants were familiar with harm reduction strategies, mainly discussing specific safer use practices or tools such as sterile syringes, naloxone, and medications for OUD rather than viewing harm reduction as a philosophical approach. For participants not familiar with the term “harm reduction,” we provided a brief definition after which participants often realized that they actually were familiar with the concept. For example, when asked about the concept of harm reduction one participant responded, “What’s that?” [18] but after clarification said that they had heard of and used principles from harm reduction. While not asked explicitly about the applicability and acceptability of harm reduction practices for themselves versus for others, participants naturally drew this distinction. This theme explores participants attitudes towards the use of harm reduction practices for their own health and safety. Many participants routinely incorporated safer use techniques in their own lives while others were less sure about the utility of harm reduction strategies for themselves.

Some participants embraced a harm reduction approach to drug use, finding such an approach applicable and acceptable for themselves. One participant shared, “I always practice using new needles. Usually, I’m with somebody and we have naloxone onsite.” [2]. Recognizing that abstinence is not a feasible or desirable outcome for everyone, some participants noted the utility of harm reduction for themselves in case their aspirations for recovery did not go as planned. One participant planning to abstain from drugs after hospitalization, though aware of the possibility of returning to drug use, endorsed being prepared with harm reduction supplies: “Am I gonna say that I’m never gonna use again? I would never say that because anything can happen. But do I want to not use? Absolutely. But I’d willing to take [harm reduction supplies] that people [offer].” [4].

Another group of participants was unsure if harm reduction practices were applicable to them if they did not inject drugs. For example, one participant shared that “I don't do needles. I'm a pill popper myself.” [7]. When asked about harm reduction strategies, another participant responded, “Let me clear–I don’t have no IV problem.” [2]. In these cases, participants perceived lower risks associated with their substance use because they did not inject and drew a distinction between general support of harm reduction strategies and the applicability of these practices to them as individuals.

The acceptability of some harm reduction interventions provided by members of the care team in the hospital setting was mixed, especially among those in or planning for abstinence following discharge. In particular, this was true for sterile syringes and other harm reduction strategies aimed at reducing infectious disease transmission that were perceived as running counter to their goals for abstinence. One participant stated that they were opposed to accepting syringes because they were in recovery: “I wouldn't even take [syringes] at this point, I would tell them I don't need it, so why would I even take it? I wouldn't.” [9]. A few participants were unclear if syringes were congruent with their goals and concerned that they may serve as a trigger for substance use, with one stating “That’s gonna make me think more along the lines of picking up again rather than not having those things in my house.” [14]. Another participant shared that being offered syringes “…would put me in a relapse mode.” [20].

Overall, participants varied in their perceptions of the relevance of harm reduction practices for their own lives. Some were candid in their desire to incorporate harm reduction strategies for their own health and safety; others did not consider them relevant in their lives, often because they did not inject drugs or because they were in recovery. Some participants acknowledged the risk of return to substance use as a risk for themselves despite their intentions to remain abstinent following discharge and were open to the relevance of harm reduction practices in that context.

### Theme 2: Applicability and Acceptability of Harm Reduction Practices for Others

Given that many participants drew distinctions between harm reduction practices for themselves versus for others, this theme includes discussion of participants’ attitudes about harm reduction practices for others. Participants defined harm reduction for others broadly, describing a range of interventions from distribution of basic supplies like clothing to interventions to reduce overdose and prevent infectious disease transmission. Supplies to meet basic needs (e.g., clothes, bus fare) were acceptable to participants for others. As one participant noted, “Make sure that they have viable shoes and socks and underwear and T-shirts and bus fare… give them a card that they can get on the [subway] with or the trolley or the bus. Or they can get something to eat.” [2].

Participants found supplies to prevent overdose applicable and acceptable for use on others. Naloxone distribution was perceived as an acceptable and expected component of treatment regardless of intention to remain abstinent in the future, often citing the fact that it could be used to assist others. When asked how they would feel if they were given naloxone upon discharge, one participant explained that “I don’t abuse [drugs], so I wouldn’t need Narcan for myself, but I can have it for somebody else.” [17]. This participant had a similar view of fentanyl test strips, finding them not applicable for themself but acceptable for others, stating “I wouldn’t need [fentanyl test strips], but the test strips is definitely needed [for others] to stay safe.” [17]. Another participant not currently using drugs shared something similar, “Narcan is always good to have in case you see somebody that’s in trouble” [20]. Another participant who found naloxone acceptable for others stated, “There's always Narcan. Yeah, it's always got a Narcan in your pocket because you never know [when you can help someone else].” [3] Overall, the majority of participants expressed comfort in accepting supplies they perceived to be for use on other people regardless of their own individual plans and many felt empowered to help others in active use with these interventions.

While harm reduction supplies to meet basic needs and naloxone were nearly ubiquitously acceptable to patients, some participants were more hesitant about syringes and other supplies to facilitate safer drug administration. As one participant shared, “Well, syringes I wouldn’t want because I don’t have the plans on using them. But the Narcan, yeah, because you never know, you might see a fellow or a young lady falling out from it and have to flip them on their side and give them the Narcan.” [8]. Relatedly, one participant simultaneously noted discomfort with harm reduction for others and its necessity, stating, “It bothers me that [harm reduction] has to be the answer. But then again, it’s like telling your kids not to have sex. You have to give them the condom and tell them what happens.” [9]. Many participants recognized that having a broad menu of harm reduction supply offerings (e.g., naloxone, syringes, pipes, straws, tourniquet, alcohol wipes, fentanyl test strips) would be helpful for others, if not specifically for themselves. However, some felt less comfortable with distribution of syringes and other supplies that they associated with their own ongoing drug use.

### Theme 3: Perceptions of Harm Reduction Conversations

We explicitly asked all participants if their hospital care team discussed harm reduction strategies or how to stay safe if they returned to substance use following discharge, and we found that discussions between the care team and patients about drug use were rare. It is worth nothing that while participants differentiated how they felt about the applicably and acceptability of harm reduction practices (e.g., use of naloxone, use of syringes) for themselves compared to others, most (though not all) participants responded to queries about harm reduction education in the hospital specifically for themselves. There was a range of reactions to the idea of harm reduction conversations in the hospital, with some participants supportive and others more hesitant, and responses were strongly influenced by the context of the discussion.

The limited clinical discussions about harm reduction that were reported by participants often happened during clinical history-taking, rather than being initiated by the care team for the purposes of exploring patient goals about drug use. For example, one participant shared that hospital providers “always ask you questions that I think are weird, but I just answer them. People ask do you share needles? Do you lick the tips? Do you use bleach? Do you dip them in bleach? Stuff like that. So, I’ve been asked them questions in the hospital every time I’ve come.” [7]. In the absence of conversations being initiated by the care team, one participant shared their history of drug use with their care team out of fear that the care team would make inappropriate clinical decisions without that information: “They wouldn’t ever know nothing about [my history of using drugs]. I mentioned it. The only reason I mentioned it was I thought it might have had something to do with my [clinical] situation.” [2]. Both of these participant reflections point to clinicians’ lack of understanding about drug use and harm reduction. They also reveal a missed opportunity for clinicians to engage with patients about harm reduction and drug use in a way that is meaningful for patients.

#### Support for harm reduction conversations in the hospital

Despite the fact that most participants reported a lack of discussion about harm reduction strategies with their care team, many patients thought the hospital setting was a suitable place for harm reduction conversations. One patient whose care team did not talk to them about drug use stated, “I’m willing to learn anything. And any kind of advice or something, you know, I’m willing to hear any of it.” [5]. Another participant shared that “I’m very familiar with [harm reduction practices] myself. But it still would have been nice to hear it again.” [20]. One participant shared a desire for harm reduction education and open discussion about drug use, stating “I would feel safe. You know, I would love that.” [14]. For these participants, open discussion did not happen, but they felt that it could have facilitated a supportive and open dynamic with the care team.

Some participants supportive of hospital-based harm reduction education highlighted the importance of tailoring such offerings based on individual goals and factors, something that is core to the harm reduction philosophy. One participant noted the contextual considerations for having these conversations based on the length of hospitalization, stating “For me, I’ll be in the hospital for over a month. So, my mind is not using. So, if they offered [harm reduction supplies] to me, I would say no. If I was in the hospital for a week, and I know I’m gonna use again, all right, give it to me.” [8]. One participant who reflected on the importance of tailoring hospital-based harm reduction conversations both for themself and for others stated: “I would suggest that they’d let [me] dictate [my] own life, like everybody should be able to dictate their own life and what direction they want they life to go into and what they want to do.” [17]. These participants conveyed the importance of patient autonomy in care and harm reduction education that is concordant with patient goals.

#### Hesitancy around harm reduction conversations in the hospital

Other participants were hesitant to have conversations about harm reduction strategies for themselves with their care team during hospitalization. Some noted discomfort about bringing substance use up to their care team because of fears about how this would be perceived. As one participant noted, “Once you’re in the hospital, I can’t see why they would ask me that because I’m here, it’s supposed to be end game. So, why tell me how I’m supposed to inject if I’m trying to stop injecting. It’s like a catch-22.” [7] Others expressed ambivalence about discussing a potential return to substance use because they were not planning to return to use substances following their hospitalization. As another participant shared: “They know that I’m trying to get out of that life. So I don’t think that they think that I need [harm reduction] anymore.” [6]. While some participants were aware of the possibility of returning to drug use after discharge, those who intended to remain abstinent from substance use after discharge were often more hesitant about the idea of harm reduction teaching and distribution of supplies in the hospital because of their optimism for recovery and perception that harm reduction supplies were not applicable to them.

## Discussion

In semi-structured interviews with hospitalized patients with OUD, we identified a broad range of applicability and acceptability that participants had about receiving harm reduction supplies and education in the hospital setting. Specifically, we noted differences in how participants viewed the applicably and acceptably of harm reduction practices that they perceived as intended to help others (e.g., naloxone) versus intended to help themselves (e.g., syringes) as well as the importance of tailoring discussions to the goal and context of an individual patient.

While participants reported that harm reduction conversations between clinicians and patients were rare, we found that discussions about harm reduction strategies were acceptable to most participants as long as discussions were consistent with their individual goals for their substance use. Some participants, however, brought up a tension between receiving harm reduction supplies from their care team, which implied ongoing drug use, and their desire for treatment and recovery. Social desirability bias may also nudge patients to endorse goals of reduced use or abstinence because they think that's what their care team wants to hear. This is unsurprising given the traditional emphasis on abstinence in the medical treatment model. The medical model also uses a framework where clinicians are the deliverers of care and patients—in this case, PWUD—are the receivers of care. Additionally, the medical model has sometimes proved hostile to PWUD by operating within a highly individualistic framework not generally accepting to patients’ decisions to continue drug use [29]. In this context, when PWUD are offered harm reduction supplies during hospitalization, they may perceive that the supplies are intended for use on themselves and imply an expectation of ongoing substance use. Accordingly, it is logical that patients would not feel comfortable openly accepting harm reduction supplies they view as for their own use as this implies that they will continue to use, which may be at odds with their own goals or the goals their perceive their care team has for them.

Community-based harm reduction organizations have largely succeeded at promoting a culture of open and honest communication about substance use, success that is at least partly due to a model of harm reduction that is more focused on community safety rather than individualism and views PWUD as people with the capacity to help others [30]. While hospitals are fundamentally different than community organizations, there may be opportunities for hospitals to learn from community organizations about how to integrate harm reduction strategies. Simultaneously, there must be recognition that much of the success of community-based harm reduction models is intrinsic to their setting and their mutual aid approach. Given that an overhaul of current hospital care delivery is unlikely, we must work within the current paradigm of hospital care. Our work provides an important look at patient perspectives on this topic and uncovers some of the ways we might integrate concepts from a harm reduction approach within a traditional medical model.

The differences between a hospital model and a harm reduction approach may also explain our finding that participants were more comfortable with supplies *perceived* to be used on others. This is consistent with existing evidence that naloxone is nearly universally acceptable to patients [31]. Most participants expressed interest in continuing to abstain from substances after discharge, perhaps because they feel that their medical care is contingent on it. Participants, however, were less open to harm reduction interventions that they likely *perceived* as intended for their own use (e.g., syringes), and this hesitancy was amplified if a person was planning for abstinence post-discharge. This phenomenon, however, may not be true in other contexts, like community-based harm reduction programs, where secondary distribution of syringes through PWUD is common and PWUD are often empowered to extend their own expertise or supplies to care for others [1]. Similarly important to note is that carrying naloxone is not always for other people—someone else could also use it on the carrier—though participants accepting naloxone from their care team most associated its utility for use on others.

As well as being core to harm reduction, understanding each patient’s goals for care is a key part of providing high quality hospital care. While we do not have specific information on conversations that took place between patients and their care team, few participants in our study reported having goal-eliciting conversations about their substance use, and conversations—when they did occur—were largely limited to history taking rather than education or intervention delivery. Hesitation from the care team to engage in harm reduction conversations may arise from limited knowledge and comfort with harm reduction and substance use, engrained stigma towards drug use, limited time, and competing clinical demands [32–36]. Some participants found harm reduction not applicable to them because they were not injecting drugs. It is worth noting, however, that many harm reduction practices are appropriate and useful for those using drugs by other means (by mouth, snorting, smoking) [37]. Such efforts are particularly important given the context of drug use present in Philadelphia and many parts of the country [38], including the adulteration of fentanyl in the drug supply and increasing polysubstance and stimulant use.

The omission of meaningful conversations about how to stay safe should a return to substance use occur following discharge (e.g., reduce use to prevent overdose due to lowered tolerance) reveals a vital difference in philosophy between the disciplines of medicine and harm reduction when it comes to substance use. A harm reduction approach elevates individual autonomy around substance use and the reduction of substance-related harms, but this approach is often foreign or uncomfortable for clinicians. Also, patients may be motivated by an abstinence-based culture of recovery in the hospital and not open to harm reduction education. Future work may need to focus on developing harm reduction education or interventions that acknowledge the complexity of substance use and the hope for recovery while acknowledging the possibility of return to use. While there are differences between harm reduction and medical models, there are approaches to guide clinicians in “difficult” conversations with patients about from everything ranging from diabetes management, sexual health, to death and dying [39–42]. In the field of palliative care, approaches have been developed for eliciting patients’ goals and preferences and preparing for the worst-case scenario while remaining hopeful for recovery. Hospitalization is too often a missed opportunity to meaningfully intervene regardless of patients’ treatment goals, and future work should start with educating clinicians about the continuum of interventions for drug use including both treatment and harm reduction strategies.

While having frank conversations about drug use is important, we must ensure that poorly executed conversations with patients about drug use do not re-traumatize or further disenfranchise patients. It is possible that members of the traditional care team are not the best people to initiate conversations about harm reduction. Is it essential to consider what members of the care team are best suited to deliver harm reduction teaching, especially given notable variation in how open clinicians are to harm reduction. It is also possible that creating a guide to harm reduction conversations for generalists could be effective, affirming and patient centered. While more research is needed to understand and inform these conversations, we also must learn from outlier US hospitals in Boston and San Francisco that have successfully incorporated harm reduction in hospital settings [4, 23]. Hospital-based harm reduction models in Canada also serve as important examples for US hospitals that lag regarding the use of evidence-based harm reduction. For example, a Vancouver hospital opened an overdose prevention site in 2018 that allows hospitalized patients to safely inject drugs under nursing supervision, as well as access harm reduction supplies and drug testing services [43]. Also in 2018, a hospital in Alberta embedded supervised consumption services which, in addition to the supervision of drug use, provides safer use education and sterile supplies [44]. These models prevent overdose, improve safety, and engage patients in addiction treatment [43, 44]. Though less comprehensive, one promising model emerging in the US is the integration of peers in recovery in the hospital [45]. Perhaps these individuals, with a combination of lived experience of substance use and recovery along with formal training, could better converse with patients about their substance use goals, although depending on the perspective of the peer, recovery and abstinence may still be presented as the ultimate goal. Another possible model could include having dedicated members of the care team like social workers or nurses, who are specially trained in having these conversations and referring people to local supplies and supports; perhaps these individuals could be embedded in an addiction consult service. Partnering with outside harm reduction organizations, as Boston Medical Center has done through implementation of a community-based harm reduction in-reach program within the hospital, also presents a possible solution for patients who do not feel comfortable talking with their providers about the possibility of return to use [23]. We must further investigate the best strategies for enabling patients to opt into safe, trauma-informed harm reduction conversations in the hospital.

Our study provides rich information about perceptions about harm reduction in the hospital. It does, however, have some limitations. Participants were predominantly white, non-Latinx, male adults receiving care at one of three urban hospitals part of an academic medical center in Philadelphia. We limited inclusion criteria to patients with OUD instead of all patients using substances, which meant that we likely missed including people who would be important candidates for harm reduction services. This inclusion criterium may also have contributed to our largely white sample, given that patients with OUD are disproportionally white [46]. While many people with OUD in Philadelphia primarily speak Spanish, only participants able to converse in English were eligible for inclusion in this study. Finally, while participants were informed that that the interviewers were not involved in participants’ clinical care, there is a chance that participants felt that what they said could impact their clinical care.

## Conclusions

Hospitalization is an important opportunity to improve the health of PWUD, regardless of their treatment goals after discharge. Offering harm reduction strategies and education is one way to do this. Our findings show that offering practical harm reduction strategies is perceived different when thought of for others verses oneself. Accordingly, harm reduction education needs to be patient-centered and informed by patient goals.

## Supplementary Information

**Additional file 1.** COREQ (COnsolidated criteria for REporting Qualitative research) Checklist.

## Data Availability

The data generated and analyzed in this study are not publicly available due to the possibility of compromising individual privacy and anonymity.

## Abbreviations

PWUD: People who use drugs
OUD: For opioid use disorder
